# The Use of Colorimetric Sensor Arrays to Discriminate between Pathogenic Bacteria

**DOI:** 10.1371/journal.pone.0062726

**Published:** 2013-05-09

**Authors:** Claire L. Lonsdale, Brian Taba, Nuria Queralto, Roman A. Lukaszewski, Raymond A. Martino, Paul A. Rhodes, Sung H. Lim

**Affiliations:** 1 Defence Science and Technology Laboratory, Porton Down, Salisbury, United Kingdom; 2 Specific Technologies, Mountain View, California, United States of America; University of Houston, United States of America

## Abstract

A colorimetric sensor array is a high-dimensional chemical sensor that is cheap, compact, disposable, robust, and easy to operate, making it a good candidate technology to detect pathogenic bacteria, especially potential bioterrorism agents like *Yersinia pestis* and *Bacillus anthracis* which feature on the Center for Disease Control and Prevention’s list of potential biothreats. Here, a colorimetric sensor array was used to continuously monitor the volatile metabolites released by bacteria in solid media culture in an Advisory Committee on Dangerous Pathogen Containment Level 3 laboratory. At inoculum concentrations as low as 8 colony-forming units per plate, 4 different bacterial species were identified with 100% accuracy using logistic regression to classify the kinetic profile of sensor responses to culture headspace gas. The sensor array was able to further discriminate between different strains of the same species, including 5 strains of *Yersinia pestis* and *Bacillus anthracis*. These preliminary results suggest that disposable colorimetric sensor arrays can be an effective, low-cost tool to identify pathogenic bacteria.

## Introduction

Certain pathogenic bacteria that present high transmission and/or mortality rates are considered high-priority bioterrorism agents because of their potential for a major public health impact. For example, *Yersinia pestis* requires only 10 organisms to infect a human with pneumonic plague [1], and *Bacillus anthracis* spores resist standard sterilization techniques, such as UV radiation, heat, and various chemicals, as well as environmental damage [2], [3], [4]. Both pathogens are highly infectious in aerosol form and listed as Category A bioterrorism agents by the Centers for Disease Control and Prevention, and both must be handled under the rigorous containment measures defined by the Advisory Committee on Dangerous Pathogens (ACDP) for a Hazard Group 3 organism. In the event of a suspected release, a rapid and reliable method to detect these pathogens would allow a faster public health response, especially if the test is inexpensive and simple to administer even in an ACDP Containment Level 3 facility.

Current bacterial identification methods can be slow and require highly trained personnel to operate sophisticated instruments that are generally too expensive, delicate, or bulky to deploy outside of a dedicated laboratory facility. Culturing remains a standard technique for identifying microbiological species, but typically requires 24 to 72 hours to support an initial diagnosis [5]. Immunoassay-based detection systems, such as ELISA, immunofluorescence, and immunoradiometric assays, are fast and easy to use, but require relatively large samples and may generate false positives from non-pathogenic bacteria in the environment [6]. Nucleic-acid amplification assays use nucleic-acid probes that are highly specific for individual pathogens and have a short assay time, but often require a clean starting sample and hours of incubation to detect and multiply the bacteria or DNA before the test. Both immunoassay and nucleic-acid-based detection strategies also require the preparation of reagents specific to the pathogen of interest in advance. All of these tests become more cumbersome when applied to particularly dangerous pathogens that must be handled under strict biological containment protocols or under the burden of protective equipment and clothing required when responding to a potential aggressive release.

Recent advances in colorimetric sensor array (CSA) technology open a promising new path to rapid and low-cost bacterial identification via analysis of the volatile metabolites that are outgassed by living microorganisms [7]. A CSA is a high-dimensional chemical sensor consisting of a two-dimensional strip of inert host material whose surface is embedded with an array of chemoresponsive reagents that individually change color when exposed to various analytes. A CSA is well suited for identifying bacterial species in a closed environment, such as a Petri dish, where headspace concentrations of individual metabolic volatile organic compounds (VOCs) range from 300 to 50,000 ppb [8]. The U.S. Army Edgewood Chemical Biological Center used gas chromatography-mass spectrometry (GC-MS) to profile the relative concentrations of the metabolic VOCs emitted by *Yersinia* and *Bacillus*, and found that the two organisms could be clearly differentiated even by visual inspection of their chromatograms [9]. In 2010, Suslick and co-workers reported that the pattern of color changes of a previous-generation CSA in response to culture headspace gas could discriminate 10 different bacterial species with 98.8% accuracy at inoculum concentrations exceeding 10^6^ CFU/plate [7]. Since then, we have substantially improved CSA sensitivity and stability by embedding indicators in a nanoporous sol-gel matrix and expanding the array of indicators [10].

Unlike methods such as GC-MS that involve component-by-component analysis, electronic-nose technologies like the CSA can classify complex chemical mixtures without ever explicitly identifying their individual components [11]. This is a major advantage when analyzing culture headspace gas, where an organism-specific metabolic signature is expected to manifest as a characteristic concentration profile entangling dozens of diverse VOCs, including aldehydes, amines, sulfides, and fatty acids [12], [13], [14], [15], [16], [17], [18], [19], [20]. Other electronic-nose technologies have attempted bacterial identification via headspace analysis, including chemiresistors, metal oxides, and fluorescent chemsensors [21], [22], [23], [24], [25], [26], [27], [28], [29], [30], [31]. However, previous gas sensors lacked the requisite sensitivity and chemical diversity to reliably detect and distinguish between different bacteria under these conditions, and most of these studies used only a single time sampling, foregoing any potential kinetic information. The current generation of CSAs can sense a variety of VOC families at concentrations ranging from low parts-per-billion to high parts-per-million [10], [32], [33]. Moreover, the CSA can be configured to include rapidly reversible reagents that allow the sensor to track temporal fluctuations in headspace VOCs throughout the bacterial growth cycle, constructing a highly discriminable kinetic profile for each species in real-time.

In this study, we examined the ability of a current-generation CSA to discriminate bacterial species and strains at substantially lower inoculum concentrations than previously reported, even under the strict biosafety protocols required to handle dangerous organisms like *Bacillus anthracis* and *Yersinia pestis*.

## Materials and Methods

### Bacterial Culturing

Bacterial strains were purchased from the National Type Culture Collection (Salisbury, U.K.) or obtained from culture collections maintained at the Defense Science and Technology Laboratory (Dstl). *Bacillus anthracis* strains Ames and Vollum and *Yersinia pestis* were handled at ACDP containment level 3. The ACDP containment level 2 bacteria, *Bacillus anthracis* UM23CL2 and *Yersinia pseudotuberculosis,* were also handled at containment level 3 to avoid any bias due to the operating environment. *Escherichia coli* and *Bacillus anthracis* were pre-cultured overnight in L-broth at 37°C with shaking at 180 rpm. *Yersinia* species were pre-cultured for 48 h at 28°C in blood agar base (BAB) broth. Cultures were diluted with PBS to achieve the desired optical density (OD_600nm_). Serial dilutions in PBS were performed to enable the desired number of CFU per plate to be delivered in 100 µL. Culture was spread over the surface of TSA +5% sheep blood agar plates (bioMérieux, Basingstoke, UK) at a concentration of 10–10^6^ CFU, and the plates were then incubated at 37°C. All bacterial strains were tested in triplicate at each concentration, except *Yersinia pestis* CO92 and *Bacillus anthracis* Vollum which had triplicate runs repeated on different days for a total of six repetitions at each concentration.

### Colorimetric Sensor Array

**Figure 1:**
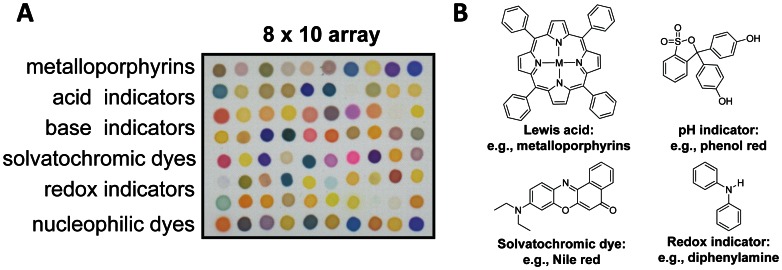
Colorimetric sensor array. A: CSA consists of 80 different chemically responsive nanoporous pigments. B: Examples of four classes of chemically-responsive dyes: (i) metal-ion-containing dyes that respond to Lewis basicity, (ii) pH indicators that respond to Brønsted acidity/basicity, (iii) dyes with large permanent dipoles (e.g., solvatochromic dyes) that respond to local polarity, and (iv) redox indicators that respond to electrochemical reaction.

CSAs were fabricated by Specific Technologies as previously reported [10], [32]. The CSA incorporated 80 different indicators covering a broad spectrum of chemical reactivity, including much stronger dye-analyte interactions than simple physical adsorption. As shown in Figure 1, the 8×10 array included metal-ion-containing dyes to sense Lewis basicity (e.g. amines), pH indicators to sense Brønsted acidity/basicity (e.g. amines and fatty acids), dyes with large permanent dipoles to sense local polarity (e.g. alcohols), metal salts to sense redox reactions (e.g. sulfides), and nucleophilic indicators to sense electrophilic analytes (e.g. aldehydes) [7]. For the complete list of indicators, see Supporting Information Table S1. To extend shelf life, the CSA was sealed inside an airtight nitrogen bag until actual use.

**Figure 2:**
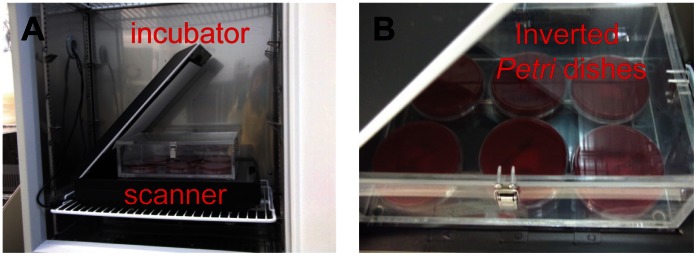
Experimental setup for solid media study. A: An incubator hosting the scanner. B: The scanner holding six Petri dishes housed within a closed plastic box. All experiments were performed using standard safety protocols in an ACDP Containment Level 3 laboratory.

Immediately after bacteria plating, the CSA was unsealed and affixed to the Petri dish cover. As illustrated in Figure 2, the CSA was clipped to a 1″×3″ microscope slide, which was mounted face-down on the inside of the Petri dish cover atop thin strips of gasket material that elevated the assembly above the cover’s surface to allow headspace gas to diffuse over the CSA. The Petri dish was placed cover-down on a flatbed scanner inside an incubator set at 37°C.

### Safety Protocol

All manipulations and incubations were carried out in an ACDP containment level 3 facility.

### Image Processing

Using an ordinary flatbed scanner (Epson Perfection V600), the CSA was imaged before initial exposure to volatiles and at 20 minute intervals after exposure, for up to 48 hours after exposure. For each image, we extracted a 240-dimensional vector of 12-bit RGB colors by taking the median of all pixels within a 20-pixel-radius circle centered in each of the 80 indicator spots. Color vectors were extracted using SpotFinder (Specific Technologies, Mountain View, CA), a semi-automated spot-finding program written in C^#^/C^++^, to manually position and orient a grid over the spot array using a mouse, automatically segment each grid cell into spot and background pixels using a global contrast threshold, and estimate each spot’s center using a flood-fill centroid of its segmented pixels. Color vectors could also be generated using ImageJ, a public-domain image-processing program available from the U.S. National Institutes of Health that was used to verify a representative subset of SpotFinder output [34].

To construct a kinetic profile for each culture, we first subtracted the color vector of a reference image of that culture from the vectors of all subsequent images, creating a time series of color differences which we smoothed with a Gaussian kernel (σ = 20 min). We ignored all images captured prior to the reference image, which was taken 4 hours after initial exposure; this choice gave most indicators enough time to finish changing color in response to the headspace out-gas of the growth medium itself. Finally, we subsampled the slopes of the smoothed color differences for each of the 240 colors at 11 evenly spaced times starting 4 h before and extending 6 h after the peak slope of a trigger indicator that was preselected for its strong and nonselective response to generic bacterial growth, creating an initial kinetic profile with 2640 features. Time to detect bacterial growth was taken to be the time of the peak trigger indicator slope. Color vectors were processed in Python using the NumPy and SciPy packages [35], [36].

### Statistical Analysis

We validated the classifier performance using leave-one-out cross-validation (LOOCV) to construct a training set for each trial. For each training set, we reduced the feature set by applying a two-tailed t-test to each feature (p<10^−5^ for species, p<0.01 for strain) for every pair of separable classes, and then trained a support vector machine (SVM) to classify the reduced feature vectors by species or strain. The trained classifier was evaluated on the kinetic profile of the corresponding test trial after projecting it onto the features selected by the training set. Validated classifier accuracy was the average misclassification rate across classes for each test trial, with 95% confidence intervals computed using Wilson’s score test. Statistical analysis was performed in R, using the stats (t-test), e1071 (support vector machine), and binom (confidence intervals) packages [37], [38], [39].

## Results and Discussion

Different bacteria emit different metabolic volatiles in a time-dynamic pattern over the course of their growth cycle. To exploit this information we examined the kinetic profile of CSA response to culture headspace VOCs [7]. When exposed to the headspace of a recently inoculated culture, the CSA’s kinetic profile generally tracked the bacterial growth curve and unfolded in four phases. First, the CSA reacted to the VOCs released by the growth medium itself. After this initial equilibration, reached within two to three hours, the CSA response leveled off because the bacteria were still in the lag phase and did not produce significant amounts of VOCs. As bacteria entered the exponential growth phase, indicator colors swung rapidly in response to the increase in headspace volatiles caused by accelerating bacterial metabolism. Finally, CSA responses converged to stable final values as bacteria entered the stationary phase. For certain bacteria, such as *Bacillus anthracis*, several indicators quickly reversed color soon after reaching a peak response, presumably reflecting diauxic shift as bacterial metabolisms adapted to depletion of preferred nutrients.

To extract a distinctive signature for each microorganism from its kinetic profile, we sampled only the window of rapid color change during the exponential growth phase. Since this exponential growth is triggered at different times in different trials, depending on inoculum concentration, we temporally aligned each trial’s kinetic profile by triggering its sample window at the advent of an inflection in slope in that trial using a preselected indicator. This is typically a CO_2_ detector whose color changed strongly and nonspecifically in response to any bacterial growth. As shown in Table 1, the sample window trigger time was concentration-dependent, and trigger times at very low concentrations (10 CFU/plate) typically lagged trigger times at much higher concentrations. Inoculum concentrations varied slightly for different species due to technical difficulty in reproducing very low concentrations, but we expect the CSA’s classification ability to be robust to such variations. The population growth rate encoded in the trigger latency was specific to each species, but this information was not considered by the classifier, because the lag due to low growth rate was inseparable from the lag due to low inoculum concentration. Once the kinetic profiles were aligned, the species-specific responses of key colorimetric indicators became relatively independent of inoculum concentration.

**Table 1:** Bacteria detection time based on strain and inoculum concentrations.

	Detection Time	
	High concentration	Low concentration
*B. anthracis* Ames	6.3 h (110,000 cfu)	7.7 h (11 cfu)
*B. anthracis* UM23CL2	7.7 h (83,000 cfu)	9.0 h (8 cfu)
*B. anthracis* Vollum	6.8 h (100,000 cfu)	7.7 h (10 cfu)
*Y. pestis* CO92	18.0 h (200,000 cfu)	30.0 h (22 cfu)
*Y. pestis* Java 9	15.0 h (87,000 cfu)	24.0 h (9 cfu)
*Y. pseudotuberculosis* YPIII	10.3 h (5,000,000 cfu)	16.0 h (50 cfu)
*E. coli* NCTC 12241	8.7 h (12,000 cfu)	11.0 h (120 cfu)

**Figure 3:**
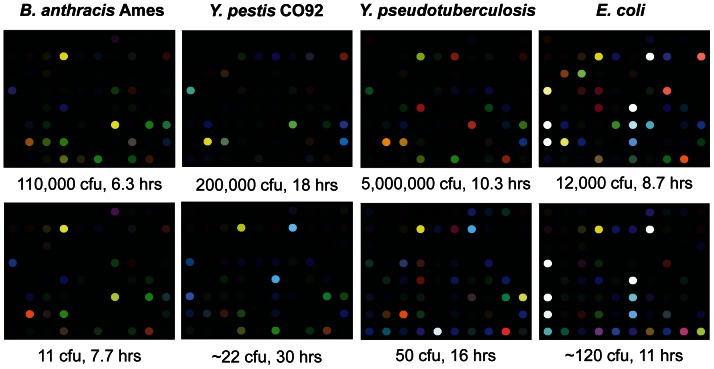
Color difference maps of *B. anthracis* Ames, *Y. pestis* CO92, *Y. pseudotuberculosis*, and *E. coli*. Each color represents the difference between the indicator color intensity measured before exposure and the intensity measured at the indicated detection time for each species. For visualization purposes, color difference maps are expanded from 4 to 8 bits per color (RGB range of 0–15 expanded to 0–255) [33].

**Figure 4:**
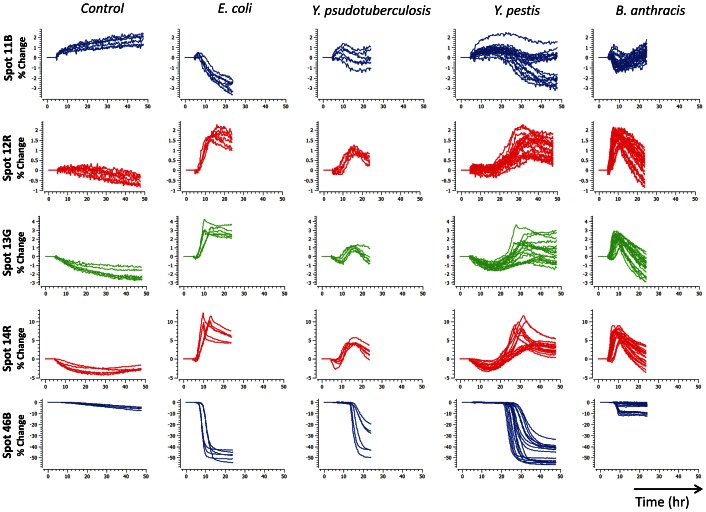
Species time response profiles. Selected time response profiles of four different bacterial species at both low and high concentrations. At least six trials were collected per species.

**Figure 5:**
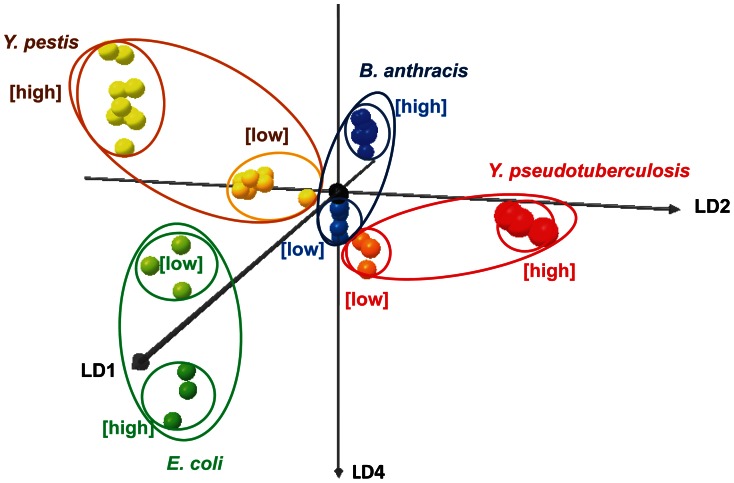
Species concentration trajectories in LDA space. Trajectories for each species from low to high concentration are separable in LDA space, suggesting that species can be identified independent of inoculum concentration. Six control trials (black) are located at the origin.

Either the color difference maps or the kinetic profiles could be used to identify bacteria. All four bacterial species tested were distinguishable by visual inspection of the color difference maps (Figure 3) or time response profiles (Figure 4). For more quantitative analysis, we used logistic regression to classify the kinetic profiles, achieving 100% accuracy on all species (95% confidence interval 94.0–100%), as described in the Materials and Methods section and summarized in Table 2. Because the classification was perfect, the confidence intervals were entirely determined by the number of samples available for each performance metric. We expect those intervals to continue to narrow as more samples are added, since the kinetic profiles are so visibly separable. To visualize the species clusters more directly, we applied linear discriminant analysis (LDA) to the reduced kinetic profiles. As shown in Figure 5, all four species could be separated in LDA-space even at low inoculum concentrations, and followed non-intersecting trajectories to increased separation at higher inoculum concentrations. Note that for ease of visualization, Figure 5 plots only 3 out of 4 linear discriminant axes, so apparent distances between nearby clusters should not be interpreted quantitatively.

**Table 2:** Species discrimination between 4 different pathogenic bacteria plus a control (95% confidence intervals in parentheses).

Species	*n*	Sensitivity	Specificity	Accuracy
*B. anthracis*	24	100% (86.2–100)	100% (90.3–100)	100% (94.0–100)
*E. coli*	6	100% (61.0–100)	100% (93.4–100)	100% (94.0–100)
*Y. pestis*	18	100% (82.4–100)	100% (91.6–100)	100% (94.0–100)
*Y. pseudotuberculosis*	6	100% (61.0–100)	100% (93.4–100)	100% (94.0–100)
Control	6	100% (61.0–100)	100% (93.4–100)	100% (94.0–100)
Total	60			100% (94.0–100)

**Figure 6:**
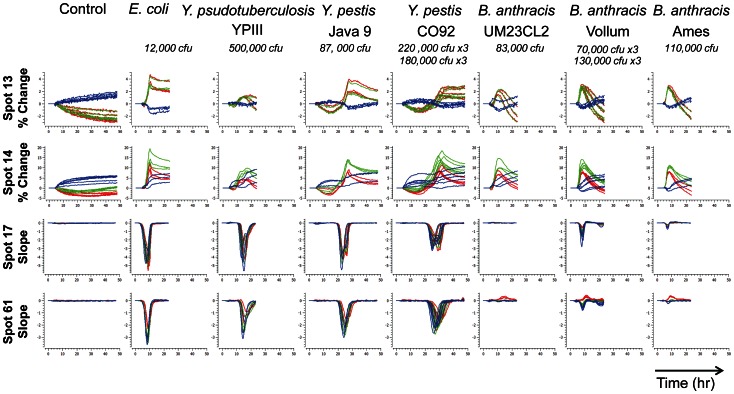
Strain time response profiles at high concentrations. For each trial, red, green, and blue lines plot either the percent change in each color from its initial intensity or the rate of the color change. Trial duration varied depending on species growth rate.

**Figure 7:**
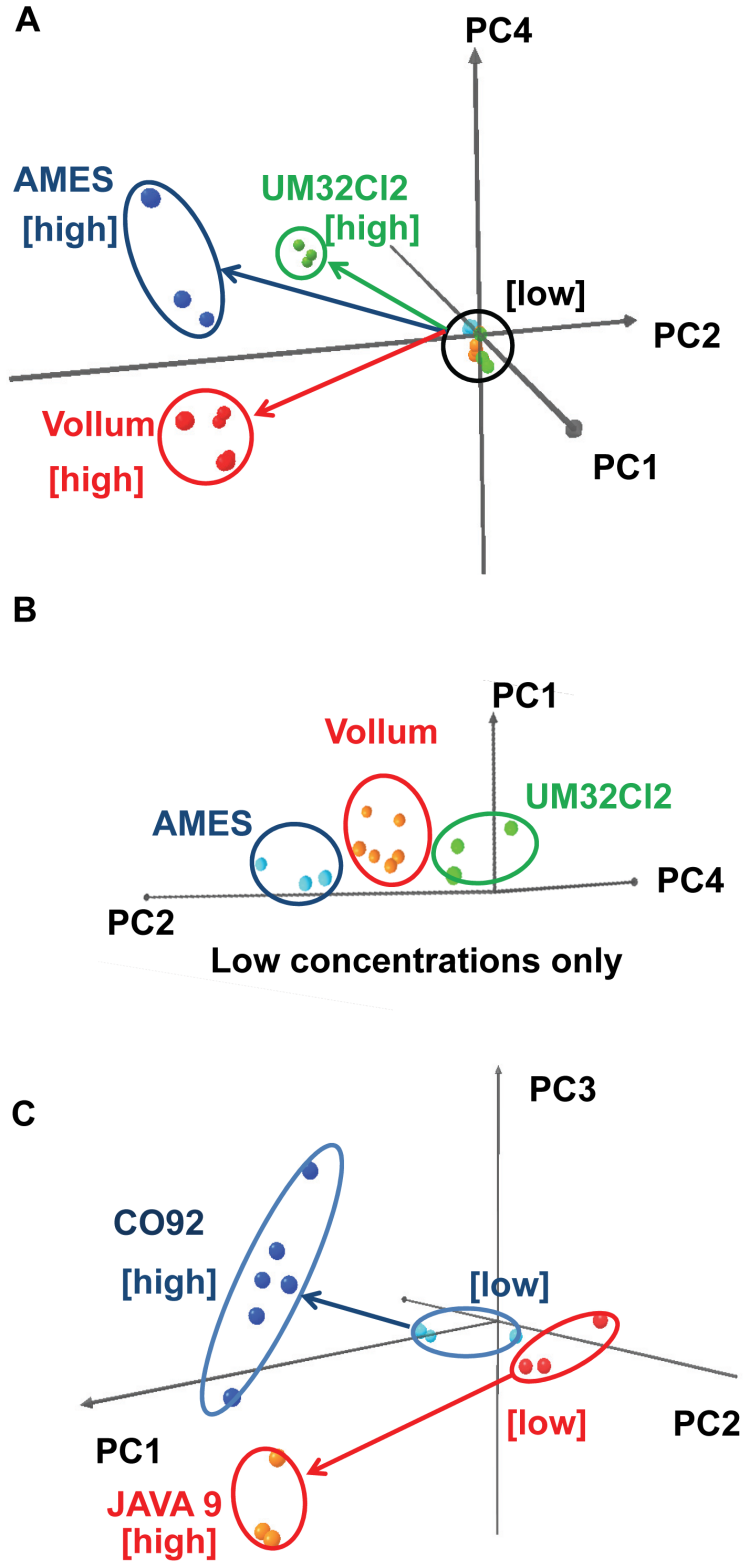
Strain separation in PCA space. A: PCA plot separating three different strains of *B. anthracis.* B: Expansion showing the separation among different strains at low inoculum concentrations. C: PCA plot for two different strains of *Y. pestis*.

Once a bacterial species has been identified, our data suggest that the CSA can further discriminate individual strains within that species (Figure 6). We successfully differentiated three different strains of *Bacillus anthracis* and two different strains of *Yersinia pestis*. For both species, the separations among strains are obvious even by visual inspection of the time response profiles of the raw color differences or slopes, as shown in Figure 6. At lower inoculum concentrations, strains are harder to pick out by eye from the time response profiles, but they can still be separated in principal-component space as shown in Figure 7. Strain classification accuracy was nearly perfect, as summarized in Tables 3–6, with the only miss being a single misclassification of one sample of *Bacillus anthracis* Vollum as *Bacillus anthracis* UM23CL2, out of 24 samples of 3 strains with initial inoculum concentrations ranging from 10 CFU to 100,000 CFU. Time to detect the presence of bacteria depended on species and concentration, ranging from 6 h at higher concentrations to 8 h for lower concentrations for *Bacillus anthracis*. Both species and strain could be identified 6 h after detection.

**Table 3:** Strain discrimination within *B. anthracis* and *Y. pestis*. (95% confidence intervals in parentheses).

Strain	*n*	Sensitivity	Specificity	Accuracy
*B. anthracis* Ames	6	100% (61.0–100)	100% (61.0–100)	100% (86.2–100)
*B. anthracis* UM23CL2	6	100% (61.0–100)	94.4% (74.2–99.7)	95.8% (79.8–99.8)
*B. anthracis* Vollum	12	91.7% (64.6–99.6)	100% (75.6–100)	95.8% (79.8–99.8)
Total	24			95.8% (79.8–99.8)
**Strain**	***n***	**Sensitivity**	**Specificity**	**Accuracy**
*Y. pestis* CO92	12	100% (75.6–100)	100% (61.0–100)	100% (82.4–100)
*Y. pestis* Java9	6	100% (61.0–100)	100% (75.6–100)	100% (82.4–100)
Total	18			100% (82.4–100)

**Table 4:** Confusion matrices of observed labels (row) and predicted labels (column) for species discrimination.

	Control	*B. anthracis*	*E. coli*	*Y. pestis*	*Y. pseudotuberculosis*	Total
Control	6	–	–	–	–	6
*B. anthracis*	–	24	–	–	–	24
*E. coli*	–	–	6	–	–	6
*Y. pestis*	–	–	–	18	–	18
*Y. pseudotuberculosis*	–	–	–	–	6	6
Total	6	24	6	18	6	60

**Table 5:** Confusion matrices of observed labels (row) and predicted labels (column) for *B. anthracis* strain discrimination.

	*B. anthracis*Ames	*B. anthracis*UM23CL2	*B. anthracis* Vollum	Total
*B. anthracis* Ames	6	–	–	6
*B. anthracis* UM23CL2	–	6	–	6
*B. anthracis* Vollum	–	1	11	12
Total	6	7	11	24

**Table 6:** Confusion matrices of observed labels (row) and predicted labels (column) for *Y. pestis* strain discrimination.

	*Y. pestis*CO92	*Y. pestis*Java9	Total
*Y. pestis* CO92	12	–	12
*Y. pestis* Java9	–	6	6
Total	12	6	18

In this report, we have demonstrated that our current-generation CSA has the necessary sensitivity and selectivity to identify both bacterial species and strain via headspace gas analysis, starting at very low inoculum concentrations of about 10 CFU/plate for several strains (Table 1). Feature extraction and LDA revealed that different strains within the same species have unique VOC patterns, independent of inoculum concentration. Although the results of this pilot study are very promising, there is considerable room to improve the technology for practical and reliable application. For example, the set of colorimetric indicators should be tuned to maximize sensitivity to the metabolic volatiles released by the species of interest; conversely, growth media could be optimized to match their VOC production to the classes of volatiles to which the CSAs are most sensitive. Replacing the open Petri dish with a smaller, sealed sample chamber could speed detection time by increasing metabolic volatile concentrations at the sensor. Finally, all of these results must be validated on a much larger and more diverse library of pathogenic and non-pathogenic bacteria. Further development of this technology could ultimately lead to a mobile handheld unit as small as a cell phone that would safely sample and incubate a hazardous pathogen, track its metabolic volatile production using a cheap and disposable sensor, and automatically match its species and strain to an onboard library of pre-recorded signatures.

## Supporting Information

Table S1 List of chemically responsive indicators.
(DOCX)
