# Supplementary material for: The Use of Colorimetric Sensor Arrays to Discriminate between Pathogenic Bacteria
Source: PLoS One. 2013 May 9;8(5):e62726. doi: 10.1371/journal.pone.0062726 (PMC3650032; doi:10.1371/journal.pone.0062726)
Supplement: Table S1 — List of chemically responsive indicators. (DOCX) [file pone.0062726.s001.docx]

**Table S1.** List of chemically responsive indicators.

| **Spot #** | **Name** |
| --- | --- |
| **1** | 5,10,15,20-Tetraphenyl-21H,23H-porphine zinc |
| **2** | 5,10,15,20-Tetraphenyl-21H,23H-porphine copper (II) |
| **3** | 5,10,15,20-Tetraphenyl-21H,23H-porphine manganese(III) chloride |
| **4** | 2,3,7,8,12,13,17,18-Octaethyl-21H,23H-porphine iron(III) chloride |
| **5** | 5,10,15,20-Tetraphenyl-21H,23H-porphine cobalt (II) |
| **6** | 5,10,15,20-Tetraphenyl-21H,23H-porphine |
| **7** | Nitrazine Yellow + TBAOH |
| **8** | Methyl Red + TBAOH |
| **9** | Chlorophenol Red + TBAOH |
| **10** | Napthyl Blue Black |
| **11** | Bromothymol Blue + TBAOH |
| **12** | Thymol Blue + TBAOH |
| **13** | *m*-Cresol Purple + TBAOH |
| **14** | Zn(II) acetate + *m*-Cresol Purple + TBAOH |
| **15** | Mercury(II) chloride + Bromophenol Blue + TBAOH |
| **16** | Mercury(II) chloride + Bromocresol Green + TBAOH |
| **17** | Pb(II)acetate |
| **18** | Tetraiodophenolsulfonephthalein |
| **19** | Fluorescein |
| **20** | Bromocresol Green |
| **21** | Methyl Red |
| **22** | Bromocresol Purple |
| **23** | Bromophenol Red |
| **24** | Brilliant Yellow |
| **25** | Lissamine Green B |
| **26** | Pyrocatechol Violet |
| **27** | *o*-dianisidine + TsOH |
| **28** | *N,N′-*Diphenyl-*N,N′*-di-p-tolylbenzene-1,4-diamine + TsOH |
| **29** | 4-(4-Nitrobenzyl)pyridine + *N*-benzylaniline |
| **30** | *o*-Tolidine |
| **31** | Lithium nitrate + Cresol Red |
| **32** | 2,3-Diaminonaphthalene |
| **33** | Silver nitrate + Bromophenol Blue + TBAOH |
| **34** | Silver nitrate + Bromocresol Green + TBAOH |
| **35** | Cresol Red + TsOH |
| **36** | Disperse Orange 25 |
| **37** | Cresol Red |
| **38** | Bromothymol Blue + TsOH |
| **39** | Tetracyanoethylene |
| **40** | Reichardt's Dye + TBAOH |
| **41** | Mercury(II) bromide + *meso*-tetra(2,4,6-trimethylphenyl)porphine |
| **42** | Chlorophenol Red |
| **43** | Bromophenol Red + TBAOH |
| **44** | Diphenylamine + TsOH |
| **45** | Malachite Green |
| **46** | Bismuth neodecanoate |
| **47** | Nile Red |
| **48** | Chlorophenol Red + TBAOH |
| **49** | Methyl Red + TBAOH |
| **50** | *o-*Phenylenediamine |
| **51** | Lead (II) acetate trihydrate |
| **52** | Bromophenol Blue |
| **53** | Brilliant Yellow + TBAOH |
| **54** | Malachite Green |
| **55** | Cresol Red |
| **56** | Copper (II) neodecanoate |
| **57** | Bromophenol Red + TsOH |
| **58** | 3,3'-Dimethoxybenzidine |
| **59** | Phenol Red + TBAOH |
| **60** | Bromophenol Blue + TsOH |
| **61** | Copper (II) naphthenate |
| **62** | Nitrazine Yellow + TsOH |
| **63** | Bromocresol Green |
| **64** | Cresol Red + TBAOH |
| **65** | 5,10,15,20-Tetraphenyl-21H,23H-porphine ruthenium(II) carbonyl |
| **66** | Copper(II) acetylacetonate |
| **67** | 2,4-Dinitrohydrazine |
| **68** | Palladium (II) sulfate |
| **69** | Phloroglucinol |
| **70** | 2,7-Dichlorofluoresceine |
| **71** | Bromocresol Purple |
| **72** | Methyl Red |
| **73** | Alizarin |
| **74** | *m*-Cresol Purple |
| **75** | Nitrazine Yellow |
| **76** | Cresol Red |
| **77** | Bromocresol Green |
| **78** | Phenol Red |
| **79** | Thymol Blue |
| **80** | Bromophenol Blue |

TBAOH: 1.0 M Tetrabutylammonium hydroxide in 2-methoxyethanol

TsOH: 1.0 M *p*-Toluenesulfonic acid in 2-methoxyethanol
